# Ethambutol Partitioning in Tuberculous Pulmonary Lesions Explains Its Clinical Efficacy

**DOI:** 10.1128/AAC.00924-17

**Published:** 2017-08-24

**Authors:** Matthew Zimmerman, Jodi Lestner, Brendan Prideaux, Paul O'Brien, Isabela Dias-Freedman, Chao Chen, Jillian Dietzold, Isaac Daudelin, Firat Kaya, Landry Blanc, Pei-Yu Chen, Steven Park, Padmini Salgame, Jansy Sarathy, Véronique Dartois

**Affiliations:** a Public Health Research Institute, New Jersey Medical School, Rutgers, The State University of New Jersey, Newark, New Jersey, USA; b Antimicrobial Pharmacodynamics and Therapeutics, Department of Molecular and Clinical Pharmacology, Institute of Translational Medicine, University of Liverpool, Liverpool, United Kingdom; c Department of Medicine, Division of Infectious Disease, New Jersey Medical School, Rutgers University, Newark, New Jersey, USA

**Keywords:** *Mycobacterium tuberculosis*, ethambutol, lesion penetration, pharmacokinetics

## Abstract

Clinical trials and practice have shown that ethambutol is an important component of the first-line tuberculosis (TB) regime. This contrasts the drug's rather modest potency and lack of activity against nongrowing persister mycobacteria. The standard plasma-based pharmacokinetic-pharmacodynamic profile of ethambutol suggests that the drug may be of limited clinical value. Here, we hypothesized that this apparent contradiction may be explained by favorable penetration of the drug into TB lesions. First, we utilized novel *in vitro* lesion pharmacokinetic assays and predicted good penetration of the drug into lesions. We then employed mass spectrometry imaging and laser capture microdissection coupled to liquid chromatography and tandem mass spectrometry (LCM and LC/MS-MS, respectively) to show that ethambutol, indeed, accumulates in diseased tissues and penetrates the major human-like lesion types represented in the rabbit model of TB disease with a lesion-to-plasma exposure ratio ranging from 9 to 12. In addition, ethambutol exhibits slow but sustained passive diffusion into caseum to reach concentrations markedly higher than those measured in plasma at steady state. The results explain why ethambutol has retained its place in the first-line regimen, validate our *in vitro* lesion penetration assays, and demonstrate the critical importance of effective lesion penetration for anti-TB drugs. Our findings suggest that *in vitro* and *in vivo* lesion penetration evaluation should be included in TB drug discovery programs. Finally, this is the first time that LCM with LC-MS/MS has been used to quantify a small molecule at high spatial resolution in infected tissues, a method that can easily be extended to other infectious diseases.

## INTRODUCTION

In 2015, tuberculosis (TB) became the single leading infectious cause of death in adults, surpassing HIV (1). Ethambutol (EMB) is part of the four-drug regimen used to treat drug-susceptible TB in the initial 2-month intensive-phase of therapy. It was introduced in the 1980s, based on a series of large clinical trials by the British Medical Research Council (2, 3). These trials indicated that EMB lacked sterilizing activity but was useful in protecting against the emergence of resistance to the three other drugs: isoniazid (INH), rifampin, and pyrazinamide (3, 4).

As a cell wall synthesis inhibitor, EMB is bactericidal against replicating bacilli but has limited potency against slow-growing and nonreplicating bacteria (5), in line with its early bactericidal activity (second best after INH [6]) and reported lack of sterilizing activity. *In vitro*, the activity of EMB is modest but similar against extracellular bacteria in liquid culture and intracellular bacilli in monocytes (7) or THP-1 macrophages (8). In mouse models, EMB is mostly static, resulting in a 2-log difference in bacterial burden between untreated and treated animals after 28 days of daily therapy at the human-equivalent dose (9; our unpublished data). Globally, the pharmacokinetics of EMB are less subject to interindividual variability than those of other first-line agents (10, 11). Absorption is not impacted by food (12), and the drug exhibits low plasma protein binding and very good distribution into uninfected mouse tissues (13). There are anecdotal reports of 8- to 10-times-higher levels in the lungs of patients who underwent pulmonary surgery for conditions other than TB (14). Favorable distribution was also demonstrated in the lung tissue and cells of noninfected primates (15). In TB patients, EMB levels were similar in pleural fluid and plasma (16) and more than 20 times higher in peripheral blood mononuclear cells than in plasma (17). However, nothing is known about the distribution, absolute concentrations, and spatial partitioning of EMB in pulmonary TB lesions.

Despite its modest activity *in vitro* and in mouse models, EMB found its place in first-line short-course chemotherapy against TB because this regimen delivered the best clinical outcome among many other combinations tested at the time (2, 3). In a pivotal trial where either streptomycin or EMB was added to isoniazid, rifampin, and pyrazinamide, the proportion of patients who converted their sputum culture by 2 months was identical in both arms (3, 18), thus conferring a clear advantage to EMB, an oral drug with a less toxic profile than streptomycin. In addition, EMB is retained in the regimens of multidrug-resistant TB patients (resistant to isoniazid and rifampin) who are sensitive to EMB. Some nontuberculous mycobacterial infections, a rapidly emerging class of lung diseases, are also treated with EMB as part of multidrug regimens (19, 20). The present study was undertaken to investigate the distribution of EMB in the lung tissue and lesions of rabbits with active TB disease. Does EMB reach the various lesion types and compartments at concentrations that are sufficient to exert bactericidal and intracellular activity? Our findings provide a rational explanation for the contribution of EMB to first-line TB treatment despite its modest potency and plasma pharmacokinetics.

## RESULTS

### *In vitro* lesion pharmacokinetics.

Previous work by our group indicated that drug distribution and partitioning within lesions is a function of uptake into macrophages and binding to caseum macromolecules (21, 22). To confirm these findings and predict the behavior of EMB at the interface between the cellular rim and necrotic core of TB lesions, we measured EMB caseum binding and uptake into macrophages of different origins. The average caseum-free fraction was high, at 35 to 38% (standard deviation [SD], 4 to 5%), predicting favorable passive diffusion through nonvascularized caseum. EMB uptake was quantified in THP-1 macrophages, classically activated (M1), alternatively activated (M2), and nonactivated (M0) murine bone marrow-derived macrophages (MDM), and human MDM isolated from three independent donors, followed by activation with heat-killed Mycobacterium tuberculosis. EMB reached higher intracellular than extracellular concentrations in all cell types tested. The average intracellular-to-extracellular concentration (I/E) ratio after 30 min of drug exposure ranged between 5 and 20, with THP-1 cells showing the lowest intracellular uptake. Overall, uptake was not significantly affected by activation status (Table 1). This favorable cellular uptake predicted good penetration into cellular lesions.

**TABLE 1:** Intracellular uptake of EMB in resting and activated primary macrophages of mouse and human origin and in THP-1 cells

Infection status	Intracellular uptake in BMDM^*b*^						
	Human			Mouse^*c*^			THP-1
	Donor 1	Donor 2	Donor 3	M0	M1	M2	
Uninfected	21.96 ± 2.78	14.98 ± 2.03	10.28 ± 8.46	6.79 ± 1.72	5.18 ± 0.90	12.44 ± 3.94	5.00 ± 1.70
Infected^*a*^	19.64 ± 3.84	11.06 ± 1.55	14.02 ± 9.82	ND^*d*^	ND	ND	ND

a Infected with gamma-irradiated (inactivated) *M. tuberculosis*.

b Intracellular uptake was determined as the intracellular-to-extracellular concentration ratio. BMDM, bone marrow-derived macrophages.

c M0, resting macrophages; M1, macrophages subjected to classical activation; M2, macrophages subjected to alternate activation (see Materials and Methods).

d ND, not detected.

### EMB penetration in cellular and necrotic lesions.

**FIG 1:**
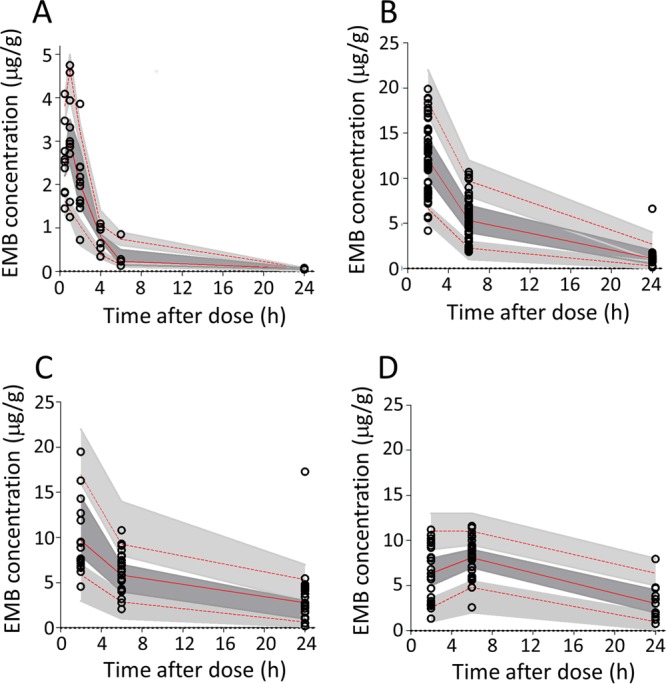
Visual predictive check for EMB concentration versus time, stratified by compartment or tissue type: plasma (A), lung (B), cellular lesions (C), and caseous/necrotic lesions (D). Observed data are shown. Top and bottom dashed lines delineate the 5th and 95th percentiles, respectively, of observed data; the solid line delineates the 50th percentile. Shaded areas encompass the 95% confidence interval for the equivalent percentiles as predicted by the final model.

To quantify the distribution of EMB in pulmonary lesions *in vivo*, a cohort of 10 New Zealand White (NZW) rabbits was infected with M. tuberculosis HN878 until they developed mature lesions (see Fig. S1 in the supplemental material), at which point they received a single oral dose of 100 mg/kg EMB at either 2 h, 6 h, or 24 h prior to lung and lesion dissection. EMB concentrations were measured in plasma collected serially from predose to necropsy and in dissected lung tissue and whole lesions sorted as either cellular or necrotic granulomas (Data set S1). Concentration-time data for EMB in plasma, lesion homogenates, and uninvolved lung were modeled using a population methodology. The final pharmacokinetics (PK) model that optimally described the data was a two-compartment structural model with first-order absorption and elimination. The parameter estimates and associated estimation of errors are summarized in Table 2. Goodness-of-fit plots are shown in Fig. S2. Significant tissue partitioning of EMB was observed; the mean values of the steady-state area under the concentration-time curve from 0 to 24 h (AUC_0–24_) measured in uninvolved lung, cellular lesions, and caseous lesions were 11.8-, 12.2-, and 8.8-fold higher than the values for the plasma AUC_0–24_, respectively (Fig. 1A to D). Distribution into necrotic lesions was slightly slower than in lung and cellular lesions, likely due to slower diffusion into avascular caseum than in well-vascularized cellular layers. Overall, EMB accumulated rapidly in lung tissue and lesions at markedly higher concentrations than measured in plasma.

**TABLE 2:** Final model PK parameter estimates for EMB in New Zealand White rabbits^*a*^

Parameter^*b*^	Estimate	Error (CV%)^*c*^
Cl (liters/h)	32.4	23.0
*V_c_* (liters)	5.71	5.60
*k*_cp_ (h^−1^)	0.500	16.6
*k*_pc_ (h^−1^)	0.210	15.6
*k*_cl_ (h^−1^)	8.10	9.72
*k*_ccell_ (h^−1^)	7.52	25.0
*k*_ccas_ (h^−1^)	2.44	17.6
*R*_cl_	3.90	24.4
*R*_ccell_	3.33	19.9
*R*_ccas_	2.41	50.2

a Rabbits received a daily dose of 100 mg/kg.

b Cl, drug clearance; *V_c_*, central compartment volume; *k*_cp_, *k*_pc_, *k*_cl_, *k*_ccell_, and *k*_ccas_, intercompartment rate constants where *c* is central compartment, *l* is lung, cell is cellular lesion, and cas is caseous or necrotic lesion; *R*_cl_, *R*_ccell_, and *R*_ccas_, compartmental penetration coefficients.

c CV%, percent coefficient of variation.

### Spatial quantitation of EMB in lesion compartments.

**FIG 2:**
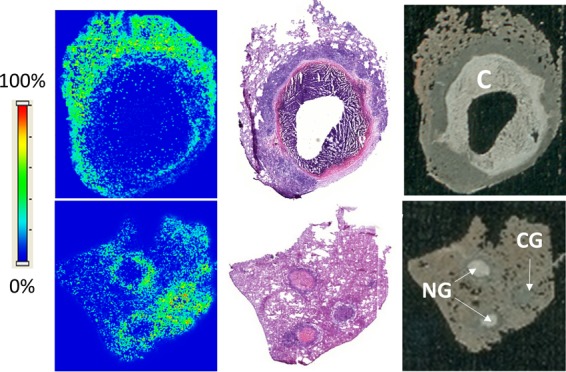
Spatial distribution of EMB in cavity (C), cellular granuloma (CG), and necrotic granulomas (NG) by MALDI mass spectrometry imaging (MSI). The left panels show ion maps of EMB ([M+H]^+^ *m/z* 205.193 ± 0.003) in a cavity (top) and two necrotic granulomas and a cellular granuloma (bottom). The intensity scale is shown on the left. The middle panel is a hematoxylin-eosin staining of the tissue section directly adjacent to the section used for MALDI MSI. The right panel shows an optical image of the section used for MALDI MSI prior to matrix application and image acquisition.

**FIG 3:**
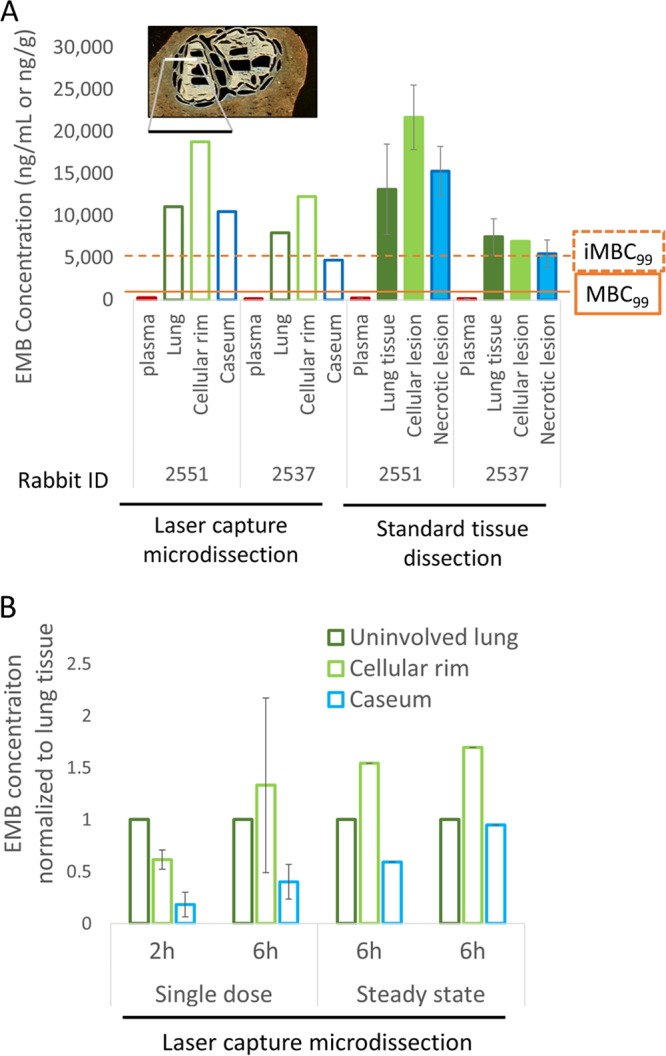
Spatial quantitation of EMB in lung and lesion compartments. (A) The left side of the panel shows absolute EMB concentrations (open bars) measured by LC/MS-MS in lung and distinct regions of necrotic granulomas, laser captured and dissected from thin-tissue sections as shown at the top of the panel (see Fig. S3 in the supplemental material for the detailed procedure). The right half of the panel (filled bars) shows data acquired by LC/MS-MS in tissue homogenates collected by standard dissection of uninvolved lung, whole cellular lesions, and whole necrotic lesions. Both rabbits 2551 and 2537 received 100 mg/kg EMB daily for 7 days, and lesions were dissected 6 h after the last dose (steady state). The minimum concentrations required to kill 99% of extracellular replicating bacilli (MBC_99_) and 99% of intracellular bacilli in macrophages (iMBC_99_) are indicated (5, 30). (B) Comparison of EMB concentration ratios between lung and cellular or necrotic lesion compartments following a single dose and at steady state. Absolute EMB concentrations were measured by LC/MS-MS in uninvolved lung, cellular rim, and necrotic core of caseous granulomas, laser captured and dissected from thin tissue sections as shown in panel A.

To visualize the relative partitioning of EMB at the interface between cellular and necrotic regions of caseous granulomas, we acquired matrix-assisted laser desorption ionization (MALDI) mass spectrometry (MS) images of EMB in necrotic granulomas and cavities obtained from rabbits that had received either single or multiple EMB doses (100 mg/kg). Although the poor ionization of EMB provided limited sensitivity, the ion maps clearly showed good distribution in the fully cellular granuloma and higher accumulation in the cellular layers than in the necrotic foci of closed caseous nodules and cavities (Fig. 2). To quantify the relative partitioning of EMB into cellular and necrotic lesion compartments, we used laser capture microdissection (LCM) coupled to liquid chromatography tandem MS (LC/MS-MS) (Fig. S3) and measured EMB concentration in microdissected caseum, cellular rim, and uninvolved lung from the lesions of rabbits that had received 7 daily doses of EMB. Lesion samples were collected 6 h after the last EMB dose or at the end of the distribution phase. This experiment was carried out at steady state since we have found that passive drug diffusion into caseum can be a slow process (21). To validate the LCM methodology, EMB was also quantified in whole dissected lesions from the same rabbits by LC/MS-MS (Fig. 3A). The results confirmed the striking accumulation of EMB in uninvolved lung and cellular rims relative to that in plasma. EMB levels measured in caseum were lower than those in the adjacent cellular layers but still markedly higher than those in plasma (Fig. 3A, empty bars). EMB concentrations measured in lung tissue and whole cellular lesions were in very good agreement with the LCM data set (Fig. 3A, solid bars, and Data set S2). The caseum-to-cellular concentration ratio was slightly higher after 7 daily EMB doses than following a single dose (Fig. 3B), suggesting that EMB diffuses slowly into caseum and accumulates in this compartment after multiple doses.

### Pharmacokinetic-pharmacodynamic (PD) target attainment at therapeutic doses.

**FIG 4:**
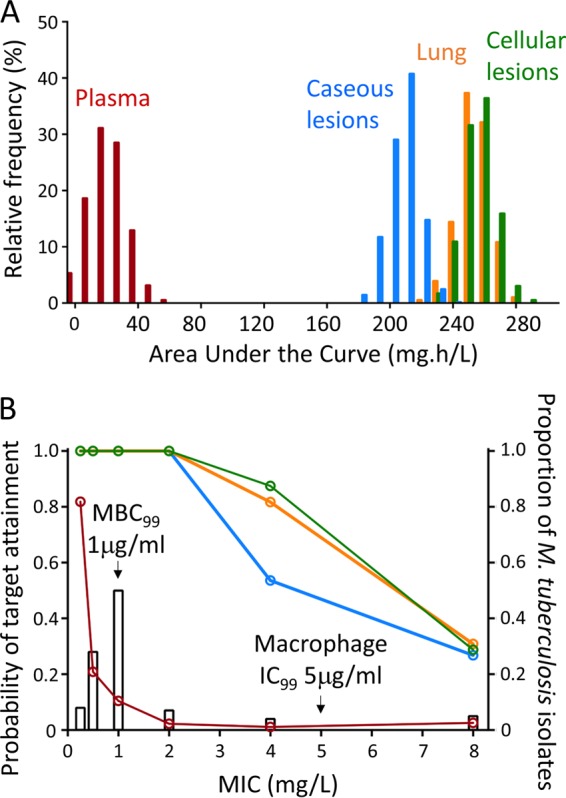
(A) Steady-state AUC_0–24_ distribution from 1,000 simulated subjects (rabbits) receiving 200 mg/kg EMB to achieve plasma AUC equivalency to adults receiving 1,200 mg, as reported by Denti et al. (10). AUC distributions are as indicated. (B) Probability of target attainment for a PK-PD target AUC_0–24_/MIC of 119 to achieve 90% of maximal kill (24) in 1,000 simulated subjects receiving a daily dose of 200 mg/kg. The right *y* axis and corresponding open bars show MIC distributions for M. tuberculosis as reported by Schön et al. (39) and Kaniga et al. (40). Red, orange, green, and blue lines indicate the probability of target attainment in plasma, lung, cellular lesions, and caseous lesions, respectively. The minimum bactericidal concentration required to achieve 99% killing (MBC_99_) (5) and the concentration required to inhibit >99% growth (IC_99_) in THP-1 macrophages (30) are indicated by arrows.

Next, we applied the model of EMB lesion penetration to determine whether adequate lesion concentrations are achieved in the lung lesions of patients receiving a standard dose. Assuming dose-proportional PK, a simulated daily dose of 200 mg/kg EMB in rabbits resulted in human-equivalent systemic exposures achieved in adults receiving daily doses of 1,200 mg daily (Table 3) (10, 12). Figure 4 shows the relative MIC coverage of EMB in the plasma and lung tissue subcompartments. At human-equivalent doses, EMB exposure in plasma did not achieve an AUC_0–24_/MIC of 119 (23, 24) in the majority of subjects across the MIC range studied. In contrast, exposures within uninvolved lung and both cellular and caseous lesions were associated with a significantly higher probability of target attainment; for isolates with an MIC of ≤2 mg/liter, the probability of target attainment in all tissue compartments exceeded 0.9.

**TABLE 3:** PK parameters of EMB in 1,000 simulated adult female New Zealand White rabbits receiving 200 mg/kg daily

Compartment	*C*_max_ (mg/liter)		AUC_0–24_ (mg · h/ml)		*T*_1/2_ (h)^*b*^	
	Estimate	Error (CV%)^*a*^	Estimate	Error (CV%)	Estimate	Error (CV%)
Plasma	5.32	18.2	24.2	5.20	4.40	6.9
Lung	22.0	25.1	282	18.1	6.54	12.0
Cellular lesion	18.4	28.1	261	12.2	8.92	18.5
Caseous lesion	17.1	14.8	212	18.8	15.7	42.0

a CV%, percent coefficient of variation.

b *T*_1/2_, half-life.

## DISCUSSION

EMB has been part of the first-line regimen since the 1980s, yet it is a moderately potent drug with little to no activity against nonreplicating bacteria. Clinically achieved concentrations in plasma are above the minimum bacteriostatic or bactericidal concentrations for a relatively small fraction of the dosing interval (11). Nevertheless, large clinical trials where EMB was replaced with either moxifloxacin or gatifloxacin, two fluoroquinolones with superior plasma-based PK-PD properties compared to those of EMB, failed to achieve treatment shortening or lower relapse rates (25, 26). Here, we show that extensive and sustained accumulation of EMB into cellular and necrotic lesions likely drives PK-PD target attainment, providing a rational explanation for the contribution of EMB to the first-line regimen. In addition to global distribution from plasma into lung lesions, we were particularly interested in the relative distribution of EMB between cellular and necrotic lesion compartments since we have shown that caseum is often the most problematic niche in terms of drug penetration (21, 27, 28). To this aim, we resorted to laser capture microdissection coupled to mass spectrometry (LCM/MS) in order to obtain spatial quantitation of EMB at the sublesional level. We show that EMB accumulates in cellular layers relative to levels in plasma but that caseum levels do not exceed those measured in plasma, suggesting that the free EMB fraction equilibrates passively between plasma and necrotic/acellular compartments. This is the first time that LCM/MS has been used to quantify a small molecule at high spatial resolution in infected tissues. We have previously proposed that drug partitioning at the caseum/cellular interface of necrotic lesions is a function of uptake into macrophages, binding to caseum macromolecules, and the calculated octanol:water partition coefficient (cLogP) (21, 22). Only drug molecules that are not taken up by macrophages and not bound to macromolecules are free to diffuse passively through nonvascularized caseum. Thus, low macrophage uptake and low caseum binding correlate with favorable partitioning into necrotic foci relative to the surrounding cellular rim, and favorable diffusion through caseum inversely correlates with hydrophobicity (cLogP). EMB (cLogP of 0.14) demonstrated high macrophage uptake and low caseum binding, which resulted in slow but substantial diffusion into caseum at steady state. Indeed, the AUC measured in cellular lesions was approximately 10 times higher than that in plasma, in line with the intracellular uptake of EMB in resting and activated primary macrophages of mouse and human origin (Table 1). This result was also consistent with the 20-fold accumulation reported in peripheral blood mononuclear cells (17). Spatial quantitation of EMB in the cellular layers and necrotic core of rabbit lesions revealed progressive diffusion into caseum, whereby the caseum-to-plasma concentration ratio was around 1 after a single dose and increased to 30 to 40 after 7 daily doses, to reach 5 to 10 μg/g in caseum throughout the dosing interval at steady state. Such a distribution pattern is reminiscent of that of rifampin (21), which is more highly bound to caseum proteins but exhibits lower uptake into macrophages. These results lend support to the emerging theory that distribution into caseum is a balance between high macrophage uptake limiting caseum penetration and low protein binding allowing free diffusion into caseum (22, 27). Overall, EMB further validates the use of these two *in vitro* assays to predict intralesional partitioning of small molecules.

In TB patients receiving 15 to 20 mg/kg, the EMB peak plasma concentration (*C*_max_) and AUC range between 2.3 and 2.8 μg/ml and 15 to 25 μg · h/ml, respectively (10, 12, 23). To predict EMB concentrations in the pulmonary granulomas of TB patients, we modeled the rabbit data set and simulated plasma and lesion concentrations at the human-equivalent dose of 200 mg/kg. The activity of EMB against intracellular M. tuberculosis ranges between 1 μg/ml to achieve a static effect (29) and 5 μg/ml to achieve cidality (30, 31). If one applies the modeled distribution of EMB in cellular lesions relative to plasma, these concentrations are expected to be achieved and maintained throughout the dosing interval in the lesions of rabbits receiving a humanized dose (see Fig. S4 in the supplemental material), with similar results in TB patients. Thus, the PK-PD metrics of EMB in cellular lesions as well as in the cellular rim of necrotic lesions point to intracellular bacilli as a major target population in human lung lesions. Since EMB is poorly active against nonreplicating M. tuberculosis bacteria (5) such as those found in caseum, it is unlikely to sterilize the caseous foci of necrotic lesions despite its favorable distribution in this compartment at steady state. Such a prediction is consistent with the lack of sterilizing activity of EMB (3, 4, 18).

EMB is used in the treatment of nontuberculous mycobacterial (NTM) disease, particularly in patients with Mycobacterium avium lung disease (19, 20), the pathology of which is similar to that of TB (32). Our findings thus extend to mycobacterial diseases in general, a growing public health threat in developed countries (33).

The following assumption and limitation must be noted. Dose-proportional exposure was assumed to simulate tissue distribution at the human-equivalent dose of 200 mg/kg for the computation of target attainment. Only a small number of rabbit lesions were subjected to laser capture microdissection and LC/MS-MS analysis following a single dose and at steady state. Larger rabbit cohorts will be required to characterize the dynamics of diffusion into caseum over time and to determine the time required for EMB to reach steady-state equilibrium between the caseous and cellular lesion compartments. While the rabbit remains a model and does not recapitulate all aspects of human pathology, we have so far observed similar patterns of lesion partitioning in rabbits and humans for rifampin, moxifloxacin, and pyrazinamide (V. Dartois and R. M. Savic, unpublished data). In summary, we have used quantitative and imaging methods to characterize the penetration and partitioning of EMB in cellular and necrotic lung lesions. While EMB plasma concentrations appear inadequate to attain proposed PK-PD targets associated with efficacy, extensive tissue partitioning results in significantly higher exposure within pulmonary tissues, which likely drives the clinical efficacy of EMB. Lesion-centric PK-PD analyses thus provide the first step toward a rational explanation for the contribution of EMB to first-line TB treatment.

## MATERIALS AND METHODS

### Macrophage uptake assays.

EMB uptake into THP-1 macrophages was determined as previously described (21).

Murine bone marrow-derived macrophages were subjected to classical (M1) or alternate (M2) activation. M1 macrophages are microbicidal and mediate resistance to intracellular pathogens, while M2 macrophages support anti-inflammatory functions such as tissue repair and tumor progression. Bone marrow was harvested from C57BL/6 mice and plated in D-10 medium (Dulbecco's modified Eagle's medium [DMEM] with 10% fetal bovine serum [FBS]) supplemented with 20% L-cell-conditioned medium (L929 cells secrete macrophage colony-stimulating factor [M-CSF] which differentiates bone marrow cells into macrophages). After 7 days, the cells (now macrophages) were harvested using a cell stripper and replated in D-10 medium with 2% L-cell medium. For M1 polarization, cells were treated with 100 U/ml of gamma interferon (IFN-γ) overnight. For M2 polarization, cells were treated with interleukin-4 (IL-4; 20 ng/ml) and IL-13 (10 ng/ml) for 2 days. The drug penetration assay was conducted in a 24-well plate format with 1 × 10^6^ cells per well. EMB was added at a final concentration of 5 μM for 30 min. Cells were then washed and lysed with distilled water prior to EMB quantitation by liquid chromatography coupled to tandem mass spectrometry (LC/MS-MS). Briefly, 100 μl of cell lysate was sampled from each well, combined with 35 μl of untreated cell lysate spiked with 1 μg/ml diclofenac as internal standard, and 15 μl of 1:1 MeOH-H_2_O. Each sample was vortexed for 5 min and centrifuged for 5 min at 10,000 rpm, and 100 μl of supernatant was transferred to a deep 96-well plate for LC/MS-MS analysis. To quantify the total number of cells/well, 50 μl of each cell lysate was removed from each well and added to a clear-bottom, black-sided 96-well plate. Fifty microliters of deionized water and 100 μl of PicoGreen (Life Technologies) were added, and the plates were incubated for 2 to 5 min, protected from light. Fluorescence was measured at 520 nm (excitation wavelength of 480 nm). Samples were blank subtracted, and cell number interpolations were made from a standard curve.

Human bone marrow-derived monocytes were isolated from the blood (purchased from the New York Blood Center) of three independent donors by Ficoll separation followed by purification with CD14^+^ MicroBeads (catalog no. 18058; Stem Cell Technologies). Approximately 2 × 10^5^ monocytes were plated in 500 μl of medium (RPMI medium supplemented with 5% FBS, 1% penicillin-streptomycin, 1% HEPES, 10% human serum, 0.01% M-CSF) in 24-well plates and incubated at 37°C with 5% CO_2_ for 2 days, after which half of the culture medium was replaced with fresh medium. After 4 more days of incubation, the culture medium was replaced with either fresh medium containing a 1:5,000 dilution of heat-inactivated M. tuberculosis (NR-14819; BEI Resources) or fresh medium in the control wells, followed by 24 h of incubation at 37°C with 5% CO_2_. (The stock of heat-inactivated M. tuberculosis was vortexed with 3-mm-diameter glass beads to disrupt the bacterial aggregates; the remaining large aggregates were allowed to sediment for 30 to 40 min, and the supernatant was frozen at −80°C in aliquots.) Next, the medium was replaced with medium containing 40 μM EMB, and the plates were incubated for 30 min. Each well was washed twice with ice-cold phosphate-buffered saline (PBS), the cells were lysed with 250 μl of double-distilled H_2_O (ddH_2_O) and incubated at 37°C for 1 h. Prior to drug quantitation, 50 μl was removed and used to quantify the viable macrophages using the PicoGreen assay as described above.

### Caseum binding assay.

The caseum binding assay was carried out by rapid equilibrium dialysis as previously described (22). Briefly, caseum was diluted 10-fold in PBS, homogenized, and spiked to give a final incubation concentration of 5 μM. The spiked matrix was placed in the sample chambers, and the buffer chambers were filled with 350 μl of PBS. The plates were then covered with adhesive seals and incubated at 37°C for 4 h on an orbital shaker set at 300 rpm. Following incubation, samples were removed from both chambers and processed by the addition of an organic solvent mixture (1:1 methanol-acetonitrile [ACN]) prior to LC/MS-MS quantification. The unbound fraction (*f_u_*) in plasma and diluted caseum was calculated as the ratio between free (buffer chamber) and total (sample chamber) drug concentrations, as shown in equation 1. A dilution factor of 10 (*D* = 10) was applied to the calculation of *f_u_* in undiluted caseum and surrogate, as shown in equation 2 (34). Recovery for each assay was calculated using equation 3. Recovery was considered acceptable between 70 and 130% (35).

(1) $$f_{u}=\frac{\left[\text{sample chamber}\right]}{\left[\text{buffer chamber}\right]}$$

(2) $$\text{Undiluted }f_{u}=\frac{1/D}{\left(\left(\frac{1}{f_{u}}\right)-1\right)+1/D}$$

(3) $$\text{Recovery}=\frac{\text{mass in sample chamber}+\text{mass in buffer chamber}}{\text{mass in sample chamber at }t=0}\times 100\%$$

### Rabbit infection and drug administration.

All animal studies were performed in biosafety level 3 facilities and approved by the Institutional Animal Care and Use Committee of the New Jersey Medical School, Rutgers University, Newark, NJ. Female New Zealand White (NZW) rabbits (Millbrook Farm, Concord, MA), weighing 2.2 to 2.6 kg, were maintained under specific-pathogen-free conditions and fed water and chow *ad libitum*. The rabbits were infected with M. tuberculosis HN878, using a nose-only aerosol exposure system as described previously (36). At 3 h postinfection, one rabbit from each round of infection was sacrificed to determine the bacterial load implanted in the lungs. At defined time points from 16 to 21 weeks postinfection, 10 rabbits received a single dose of 100 mg/kg EMB formulated in 40% sucrose by oral gavage, and 3 rabbits received 7 daily doses of 100 mg/kg EMB to reach steady state. These time points were selected to ensure that both cellular and necrotic granulomas had formed and reached a size sufficient to allow dissection of individual lesions. At 16 to 21 weeks postinfection, all rabbits have both cellular and necrotic granulomas. Cavities are occasional and were treated as necrotic lesions in this study (i.e., the caseum was not separated from the cavity wall for LC/MS-MS analysis). Blood was collected from the central ear artery of each rabbit predose and at several time points between drug administration and necropsy (typically, 0.5, 1, 2, 4, 6, and/or 24 h following drug administration). Groups of 2 to 4 rabbits were euthanized at 2 h (4 rabbits), 6 h (4 rabbits), and 24 h (2 rabbits) postdose. These time points were selected based on the plasma PK profile to capture the *C*_max_/*T*_max_ (2 h; where *T*_max_ is time to maximum concentration of drug in serum), the end of the distribution phase (6 h), and the trough, or minimum concentration (*C*_min_; 24 h). All blood samples were centrifuged at 4,000 rpm for 5 min, and the supernatants (plasma) were transferred and stored at −80°C until analyzed by high-pressure liquid chromatography (HPLC) coupled to tandem mass spectrometry (LC/MS-MS).

### Lesion dissection and processing.

The right and left lungs were removed and weighed for analytical drug measurement and histopathology. From each lung lobe, individual granulomas and uninvolved (nondiseased) lung tissue sections were dissected, sized, weighed, and recorded. Lesions weighing less than 5 mg were pooled. Special care was taken to remove the uninvolved lung tissue surrounding each granuloma. The samples collected from each rabbit were classified as uninvolved lung, necrotic or cellular granulomas, cavity wall, or cavity caseum. When necrotic granulomas were greater than 7 mm, they were dissected so that the lesion wall and the caseous material within could be stored and analyzed separately. Lesions collected for laser capture microdissection were left embedded in the surrounding tissue and snap-frozen in liquid nitrogen vapor as described previously (27). All samples were stored in individual 2-ml tubes at −80°C.

Prior to drug quantitation by LC/MS-MS, all tissue samples were homogenized in approximately (but accurately recorded) 4 volumes of phosphate-buffered saline (PBS). Homogenization of all tissue samples was achieved using a FastPrep-24 instrument (MP Biomedicals) and 1.4-mm zirconium oxide beads (Precellys). Extraction was performed by adding 180 μl of 1:1 acetonitrile-methanol containing 100 ng/ml stable deuterium-labeled EMB (EMB-d_10_) to 20 μl of plasma or homogenized tissue sample and 20 μl of 1:1 acetonitrile-water (ACN-H_2_O). The mixtures were vortexed for 5 min and centrifuged at 4,000 rpm for 5 min, separating the precipitated proteins from the extracts that were then transferred for LC/MS-MS analysis.

### MALDI-MSI of EMB in rabbit lesions.

Tissue sections (12 μm) were cut from gamma-irradiated rabbit lung biopsy specimens using a Microm HN505 N (Walldorf, Germany) and thaw-mounted onto stainless steel slides (MALDI-mass spectrometry imaging [MALDI-MSI]) or standard glass microscope slides (for hematoxylin and eosin [H&E] staining).

Plates containing tissue sections for MALDI-MSI were allowed to reach room temperature for 15 min prior to the opening of the containers. 2,5-Dihydroxybenzoic acid matrix (25 mg/ml in 60% methanol–0.1% trifluoroacetic acid [TFA]) (Sigma-Aldrich, St. Louis, MO) was applied to the tissues via a TM-Sprayer automated MALDI tissue prep device (HTM Technologies, NC) under the following optimized conditions: 0.04-ml/min flow rate, 60°C nozzle temperature, and 1.3-mm/s raster speed, with 25 passes over the tissue. EMB-d_10_ (C/D/N Isotopes, Quebec) was added to the matrix at 5 pmol/μl as an internal standard.

MALDI-MSI acquisition was performed using a MALDI linear trap quadrupole (LTQ) Orbitrap XL mass spectrometer (Thermo Fisher Scientific, Bremen, Germany) with a resolution of 60,000 at *m/z* 400 and full width at half maximum. Imaging data were acquired in full scan mode to maximize sensitivity, and drug peak identities were confirmed by acquiring several MS/MS spectra directly from the dosed tissues.

Instrument parameters were tuned and optimized on spiked EMB drug standard on stainless steel plates and control mouse lung tissue. The limit of detection (LOD) for MALDI-MSI analysis of EMB was 1 μg/g, calculated as described previously (21).

Spectra were acquired in positive mode across the mass range of *m/z* 190 to 400. A laser energy of 20 μJ was applied, and five laser shots were fired at each position (total of 1 microscan per position). The laser step size was set at 50 μm, which enabled small necrotic areas within lesions to be resolved without overlap of the laser spot on adjacent acquisitions. Thermo ImageQuest software (version 1.01) was used to reconstruct two-dimensional (2D) ion images. Normalized ion images of EMB were generated by dividing EMB [M+H]^+^ signal (*m/z* 205.193 ± 0.003) by EMB-d_10_ [M+H]^+^ signal (*m/z* 215.039 ± 0.003).

### Laser capture microdissection of rabbit tissue slices.

Tissue sections (25 μm thick) were cut from gamma-irradiated rabbit lung biopsy specimens using a Microm HN505 N (Walldorf, Germany) and thaw-mounted onto 1.4-μm-thick Leica PET membrane frame slides (Herborn, Germany) for laser capture microdissection. Tissue sections were immediately stored in sealed containers at −80°C. Adjacent 12-μm-thick tissue sections were thaw-mounted onto standard glass microscopy slides for H&E and Ziehl-Neelsen staining.

Cellular, necrotic (caseum), and uninvolved lung lesion areas totaling 5 million μm^2^ were dissected from between 3 and 6 serial lung biopsy tissue sections using a Leica LMD6500 system (Buffalo Grove, IL). Areas of cellular and caseous lesion were identified optically from the bright-field image scan and by comparison to the adjacently sectioned H&E reference tissue. Pooled dissected lesion tissues were collected into 0.25-ml standard PCR tubes and immediately transferred to −80°C. Prior to analysis, the tubes were thawed at room temperature for 30 min. Fifty microliters of extraction solution (ACN-MeOH [1:1] with 10 ng/ml Verapamil and 100 ng/ml EMB-d_10_) was added to each tube, and tubes were then sonicated for 5 min and centrifuged at 10,000 rpm for 5 min at room temperature. Forty microliters of supernatant was transferred for LC/MS-MS analysis and diluted with an additional 40 μl of MilliQ water.

Neat dimethyl sulfoxide (DMSO; 1 mg/ml) stocks for all compounds were diluted serially in 50/50 acetonitrile-water to create standard curves and quality control spiking solutions. Two microliters of neat spiking solution was added to 2 μl of lesion homogenate, and extraction was performed by adding 50 μl of extraction solution (ACN-MeOH [1/1] with 10 ng/ml Verapamil and 100 ng/ml EMB-d_10_). Extracts were vortexed for 5 min and centrifuged at 10,000 rpm for 5 min. Forty microliters of supernatant was transferred for LC/MS-MS analysis and diluted with an additional 40 μl of MilliQ water. Previously optimized LC/MS-MS parameters were used for analysis (see “HPLC-mass spectrometry method” below). The total tissue volume of each pooled sample was determined based on the surface area of the pooled sections and the 25-μm tissue thickness. A dilution factor was used to normalize the tissue volumes with the standard curve for quantification. Note that this analysis was run on 3-year-old tissue samples; despite having been stored at −80°C, EMB had partially degraded, which explains the concentrations 2- to 3-fold-lower than those measured by standard LC/MS-MS using lesion homogenates.

### HPLC-mass spectrometry method.

Neat DMSO (1 mg/ml) stocks for all compounds were serially diluted in 50/50 acetonitrile-water to create standard curves and quality control spiking solutions. Twenty microliters of neat spiking solutions was added to 20 μl of drug-free plasma or control tissue homogenate, and extraction was performed as described above. NZW control plasma treated with K_2_EDTA was obtained from Bioreclamation and used to build standard curves. Gamma-irradiated lung, lesion, and caseum samples from tuberculosis-infected NZW rabbits were used as control tissues and homogenized as described above. LC/MS-MS analysis was performed on a Sciex Applied Biosystems Qtrap 4000 triple-quadrupole mass spectrometer coupled to an Agilent 1260 HPLC system to quantify EMB in the plasma and lung samples. Chromatography was performed with an Agilent Zorbax SB-C_8_ column (4.6 by 50 mm; particle size, 3.5 μm) using a reverse-phase gradient elution with ion pairing reagents. The aqueous mobile phase consisted of 6 mM heptafluorobutyric acid (HFBA) and 10 mM ammonium hydroxide (NH_4_OH) in water, and the organic mobile phase consisted of 3 mM HFBA and 10 mM NH_4_OH in 95:5 ACN-H_2_O. Multiple-reaction monitoring (MRM) of parent/daughter transitions in electrospray positive-ionization mode was used to quantify the analytes. The MRM transitions were EMB (205.1/116.2) and EMB-d_10_ (215.10/123.20). Sample analysis was accepted if the concentrations of the quality control and standards were within 20% of the nominal concentration. Data processing was performed using Analyst software (version 1.6.2; Applied Biosystems Sciex). EMB and the EMB-d_10_-labeled internal standard were purchased from Alfa Aesar and C/D/N Isotopes, respectively.

### Pharmacokinetic modeling.

Concentration-time data for EMB in plasma, lesion homogenates, and uninvolved lung homogenates from each rabbit, determined by LC/MS-MS, were modeled using a population methodology. Data were fit to nonlinear mixed-effects models with a first-order conditional estimation method as implemented in the software NONMEM (version 7.3; Icon Development Solutions, Ellicott City, MD). Graphical, statistical, and exploratory analyses were conducted using the open-source software platform R (version 3.3.1). The Xpose (version 4.0) package, implemented within R, was used for graphical evaluations and visual predictive checks.

A structural PK model was fit to the plasma PK data. Second, a full model describing penetration into uninvolved lung, caseous, and cellular lesions was fit using an “effect compartment” approach as described by Savic et al. and Sheiner et al. (37, 38). Additive and proportional error models of residual variability were explored for plasma, lung, and lesions. Finally, all parameters were reestimated simultaneously using all available data. The statistical significance of parameter addition was judged on the basis of a log-likelihood ratio test, based on reduction of the objective function value (OFV) with an acceptance *P* value of 0.05.

The final PK model that optimally described the data was a two-compartment structural model with first-order absorption and elimination according to the following ordinary differential equations,

(4) $$\frac{dC_{a}}{dt}=-k_{a}\times C_{a}$$

(5) $$\frac{dC_{c}}{dt}=k_{a}\times C_{a}-\left(k_{\text{cp}}+\text{Cl}/V_{c}\right)\times C_{c}+\left(k_{\text{pc}}\times C_{p}\right)$$

(6) $$\frac{dC_{p}}{dt}=k_{\text{cp}}\times C_{c}-k_{\text{pc}}\times C_{p}$$

where *C* represents the amount of EMB (in milligrams) in the absorption (*a*), central (*c*) and peripheral (*p*) compartments, *V_c_* and Cl represent central compartment volume and EMB clearance, respectively, *k_a_* is the absorption rate constant, and *k*_cp_ and the *k*_pc_ are intercompartment rate constants.

(7) $$\frac{dC_{l}}{dt}=k_{\text{cl}}\times \left(R_{\text{cl}}\times \frac{A_{c}}{V_{c}}-C_{l}\right)$$

(8) $$\frac{dC_{\text{cell}}}{dt}=k_{\text{ccell}}\times \left(R_{\text{ccell}}\times \frac{A_{c}}{V_{c}}-C_{\text{cell}}\right)$$

(9) $$\frac{dC_{\text{cas}}}{dt}=k_{\text{ccas}}\times \left(R_{\text{ccas}}\times \frac{A_{c}}{V_{c}}-C_{\text{cas}}\right)$$

Equations 7 to 9 represent EMB penetration within lung and caseous and cellular lesions, where *C* represents the amount of EMB (in milligrams) in the uninvolved lung (*l*), cellular (cell), and caseous (cas) lesions. *k*_cl_, *k*_ccell_, and *k*_ccas_ are intercompartment rate constants, *R*_cl_, *R*_ccell_, and *R*_ccas_ represent penetration coefficients between the central compartment and lung or lesion, *A_c_* is the amount of drug in the central compartment, and *A_c_*/*V_c_* is the drug concentration in plasma at time *t*.

### Model validation.

The final models of EMB were validated using a nonparametric bootstrap resampling technique. A total of 1,000 bootstrap data sets were generated based on random sampling with replacement. The final model was fit to the bootstrap data, and PK parameters were reestimated. Measures of central tendency and dispersion and the 95% confidence interval (CI) for each parameter value were calculated and compared with the original parameter value's estimates.

### Simulations and exploration of humanized regimens.

Predicted AUC_0–24_ and *C*_max_ values in plasma, lung, and lesions were computed. Visual predictive checks were performed to evaluate the simulation properties of the final model and to explore tissue kinetics following administration of doses predicted to achieve human-like systemic exposures based on published data (10, 12, 23).

### Pharmacokinetic-pharmacodynamic exposure target selection.

PK-PD target indices were identified for EMB from available literature. The drug exposure index that best explains microbial kill of M. tuberculosis by EMB is the ratio of the AUC_0–24_ to the MIC (AUC_0–24_/MIC). An AUC_0–24_/MIC ratio of 119 has been associated with 90% maximal effective concentrations (EC_90_) for EMB (24). Target attainment analysis was performed based on the distribution of EMB MICs for wild-type strains obtained from published epidemiological data based on clinical isolates tested according to standardized EUCAST methodology (39, 40).

## Supplementary Material

Supplemental material
