# Supplementary material for: Ethambutol Partitioning in Tuberculous Pulmonary Lesions Explains Its Clinical Efficacy
Source: Antimicrob Agents Chemother. 2017 Aug 24;61(9):e00924-17. doi: 10.1128/AAC.00924-17 (PMC5571334; doi:10.1128/AAC.00924-17)
Supplement: Supplemental material [file supp_61_9_e00924-17__index.html]

Supplemental material 

# Ethambutol Partitioning in Tuberculous Pulmonary Lesions Explains Its Clinical Efficacy

## Supplemental material

- Supplemental file 1 -

  Data Set S1

  XLSX, 16K
- Supplemental file 2 -

  Data Set S2

  XLSX, 14K
- Supplemental file 3 -

  Figures S1 to S4

  PDF, 960K
