# Supplementary material for: Ethambutol Partitioning in Tuberculous Pulmonary Lesions Explains Its Clinical Efficacy
Source: Antimicrob Agents Chemother. 2017 Aug 24;61(9):e00924-17. doi: 10.1128/AAC.00924-17 (PMC5571334; doi:10.1128/AAC.00924-17)
Supplement: Supplemental material [file AAC.00924-17_zac009176531s3.pdf]

Ethambutol partitioning in tuberculous pulmonary lesions explains its clinical efficacy

Matthew Zimmerman [(1) €], Jodi Lestner [(1, 2) €], Brendan Prideaux [(1)], Paul O'Brien [(1)], Isabela Freedman [(1)], Chao Chen [(1)], Jillian Dietzold [(3)], Isaac Daudelin [(1)], Firat Kaya [(1)], Landry Blanc [(1)], Pei-Yu Chen [(1)], Steven Park [(1)], Padmini Salgame [(3)], Jansy Sarathy [(1)] and Véronique Dartois [(1) ‡]

[(1)] Public Health Research Institute, New Jersey Medical School, Rutgers, The State University of New Jersey, 225 Warren St, Newark, NJ 07103

[(2)] Antimicrobial Pharmacodynamics and Therapeutics, Department of Molecular and Clinical Pharmacology, Institute of Translational Medicine, University of Liverpool, UK

[(3)] Department of Medicine, Division of Infectious Disease, New Jersey Medical School, Rutgers University, Newark, NJ.

[€] contributed equally to the present work

[‡] To whom correspondence should be addressed: veronique.dartois@rutgers.edu

Supplemental figures

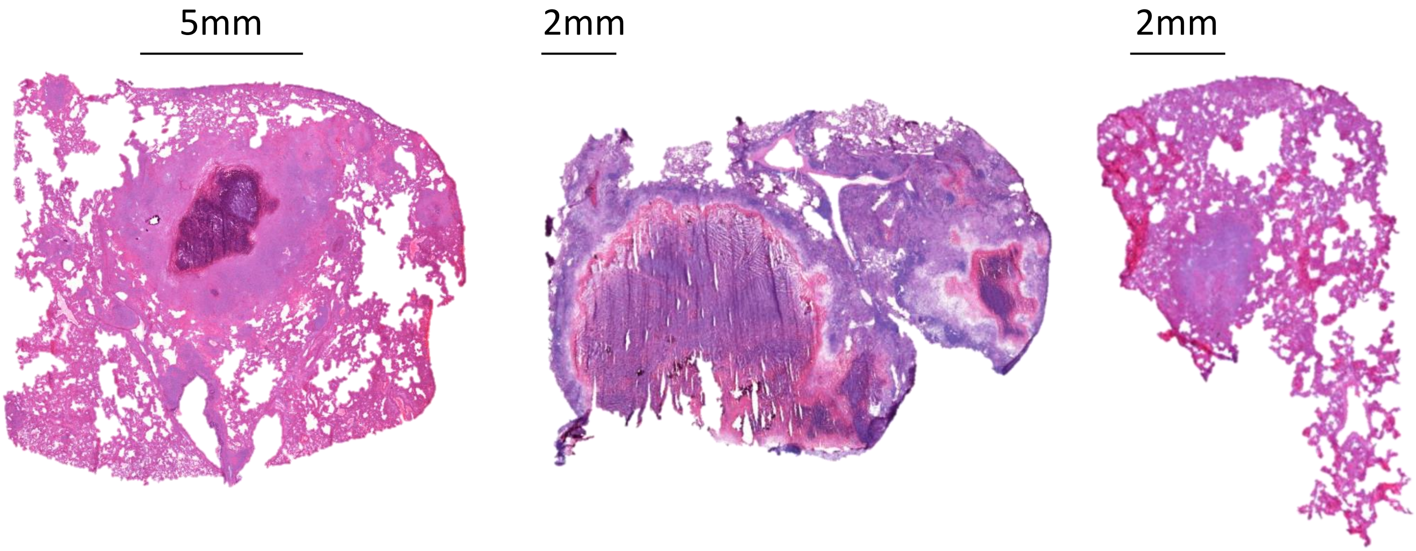

Figure S1. Typical example of a cavity, necrotic and cellular granuloma sampled in this study

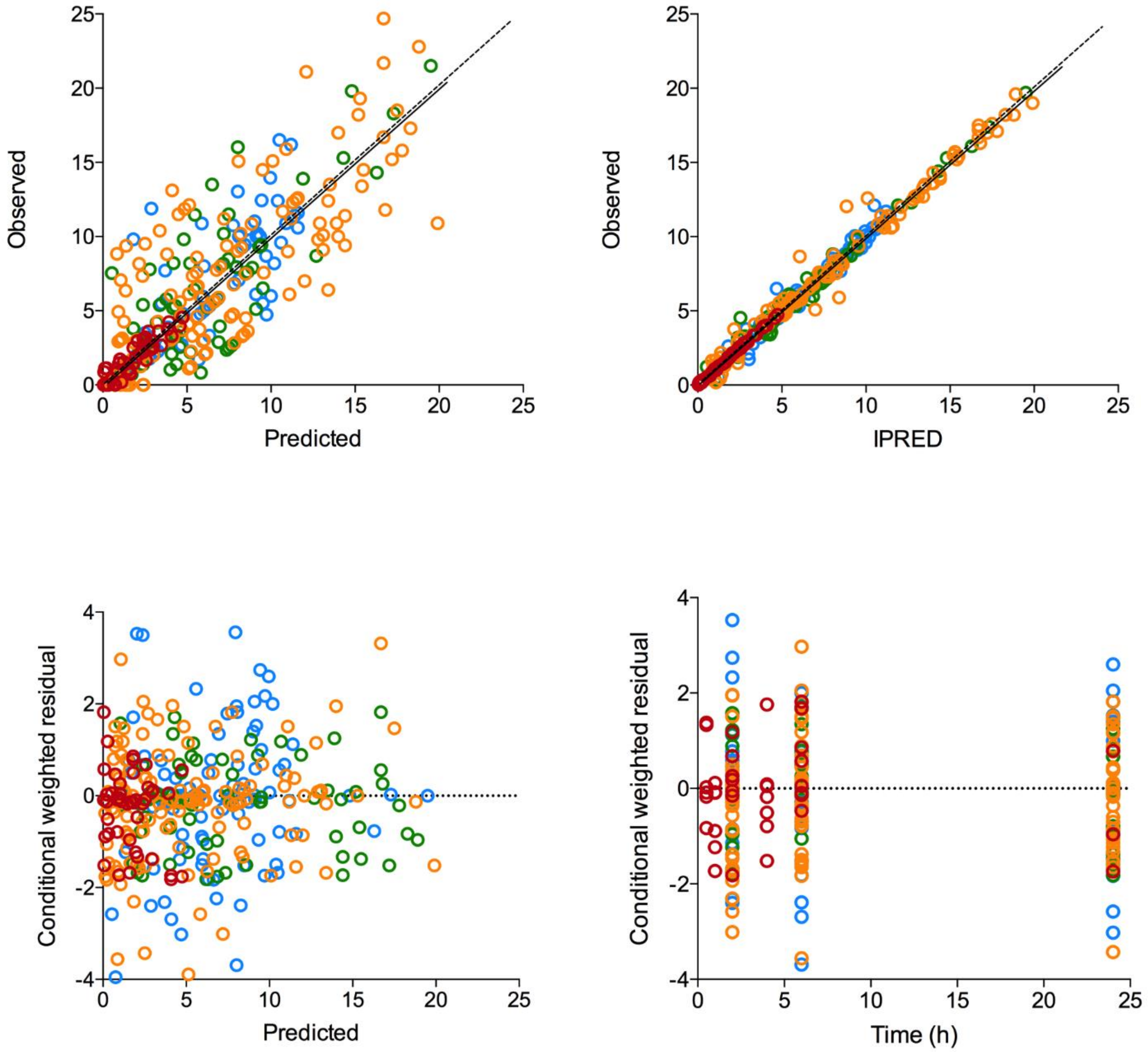

Figure S2. Goodness-of-fit plots for the final model structure. Red, orange, green and blue circles are data in plasma, lung, cellular lesions and caseous lesions, respectively. IPRED, individual predicted.

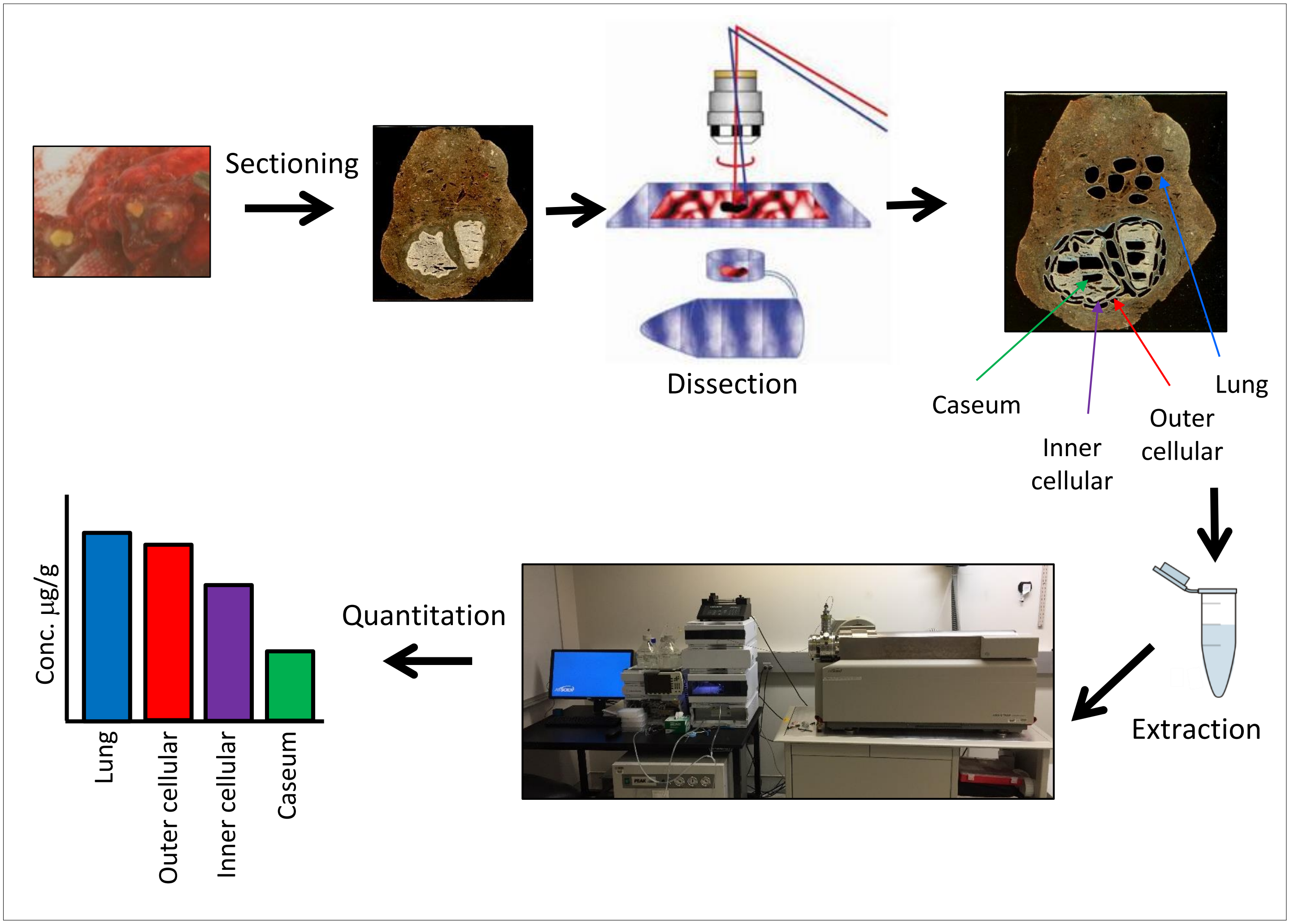

Figure S3. Schema describing the workflow of laser-capture microdissection coupled to sample analysis by HPLC/tandem mass spectrometry.

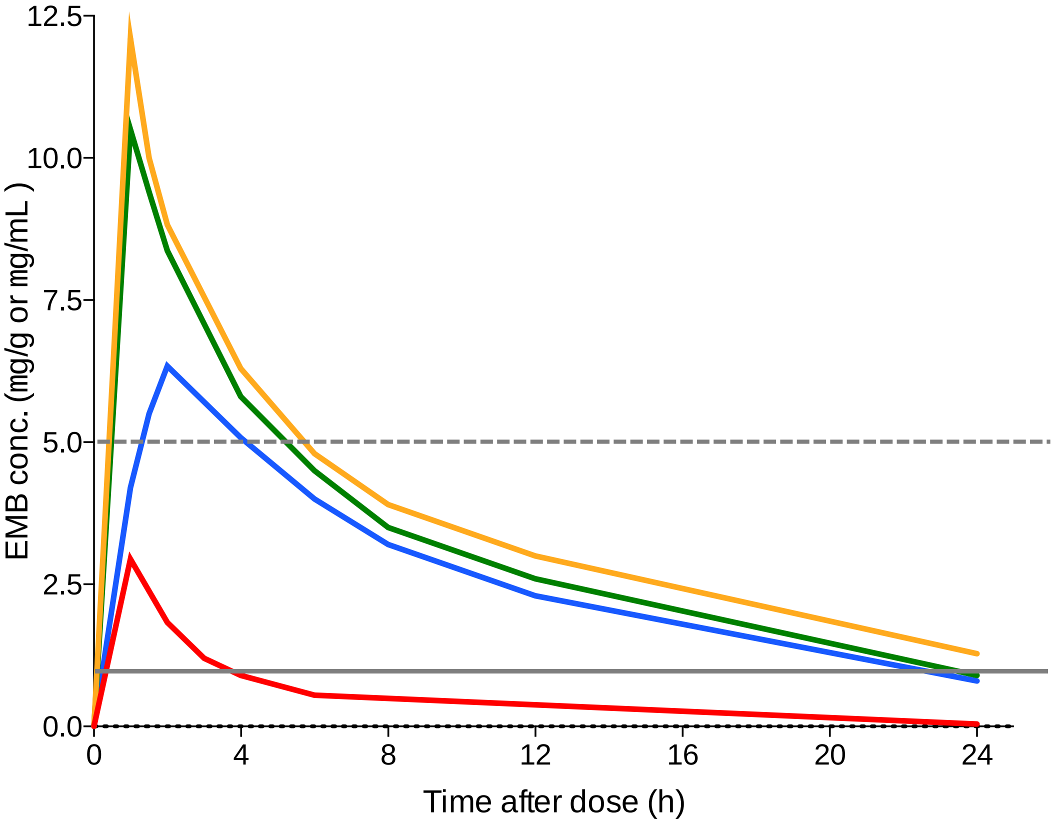

Figure S4. Simulated concentration-time profiles showing average exposures (population mean) for subjects (rabbits) receiving 200 mg/kg EMB to achieve plasma AUC equivalency to adults receiving 1,200 mg. Red, plasma; orange, lung; green, cellular lesions and blue caseous lesions. The solid grey line indicates the MIC (minimum inhibitory concentration) against extracellular M. tuberculosis bacilli as well as the minimum static concentration against intracellular *M. tuberculosis*. The dashed grey line indicates the MBC_{99} (concentration which kills 99% of replicating extracellular bacilli) and minimum cidal concentrations against intracellular *M. tuberculosis.*
